# Supplementary figures and images for: Memory impairment in chronic experimental Chagas disease: Benznidazole therapy reversed cognitive deficit in association with reduction of parasite load and oxidative stress in the nervous tissue
Source: PLoS One. 2021 Jan 5;16(1):e0244710. doi: 10.1371/journal.pone.0244710 (PMC7785227; doi:10.1371/journal.pone.0244710)

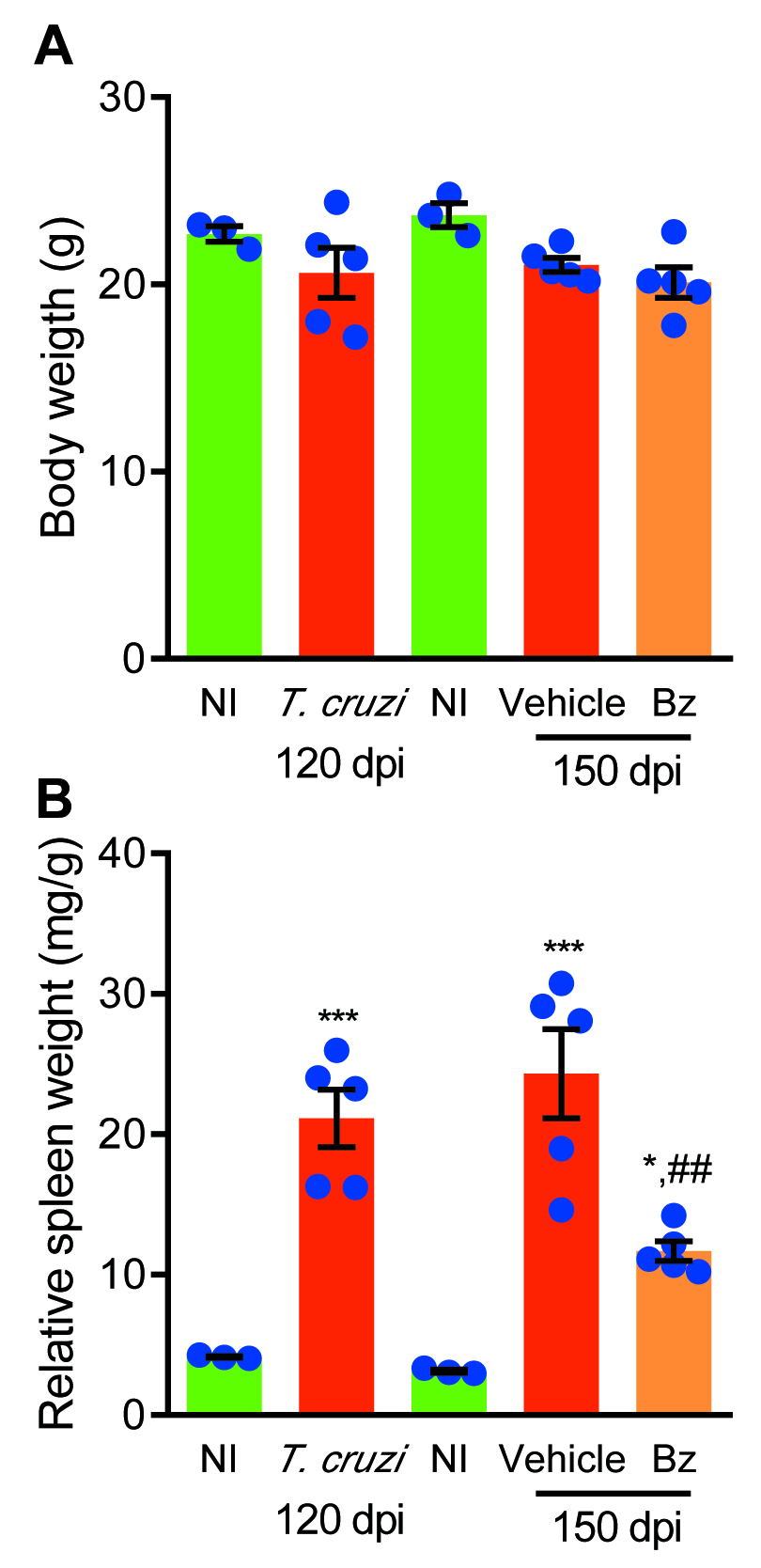

Supplement: S1 Fig — C57BL/6 mice were infected with 100 blood trypomastigotes of the Colombian strain of T. cruzi, and treated with Veh or Bz, as described in legend of Fig 3. (A) Body weight (g). (B) Relative spleen weight (mg/g). Data represent two independent experiments with 3 NI controls and 5 infected mice per group. Each circle represents an individual mouse. Data are shown as means ± SE and were analyzed using ANOVA-Bonferroni posttest. *, p<0.05 and ***, p<0.001, comparing T. cruzi-infected and NI mice; ##, p<0.01, comparing Bz-treated and Veh-treated T. cruzi-infected. (TIF) [file pone.0244710.s002.tif]
