# Supplementary material for: Memory impairment in chronic experimental Chagas disease: Benznidazole therapy reversed cognitive deficit in association with reduction of parasite load and oxidative stress in the nervous tissue
Source: PLoS One. 2021 Jan 5;16(1):e0244710. doi: 10.1371/journal.pone.0244710 (PMC7785227; doi:10.1371/journal.pone.0244710)
Supplement: Attachment — Submitted filename: PLOS review.docx [file pone.0244710.s003.docx]

Dear PLOS ONE

**Manuscript Number:** PONE-D-20-30457
**Manuscript Title:** Memory impairment in chronic experimental Chagas disease: benznidazole therapy reversed cognitive deficit in association with reduction of parasite load and oxidative stress in the nervous tissue

The authors aimed to investigate effects of Colombian type I T. cruzi strain on cognitive function using C57BL/6 mice assessed by novel object recognition task, open field test and an aversive shock evoked test. They also studied effects of Bz therapy on restoring memory loss. They successfully show that T. cruzi infection leads to impairment of memory that is rescued by Bz therapy compared to non-infected controls.

Minor revisions

1. Line 52 please define Veh-treated infected mice
2. Lines 132-135 please define the abbreviations
3. Line 202 please define NI
4. Line 360 tell the reader here how you define ‘elderly’
5. Line 364 kindly check the grammar for ‘tissues section’ sentence
6. Line 416 remind the reader how you determined ‘chronically infected mice did not display neuromuscular disorders’
7. Line 509 define ‘NF-kB- and MAPK’
8. Line 520 define ‘TNFR1’
9. Line 532 define a ‘TBARS’

I suggest engaging someone to go over minor grammatical glitches in the manuscript

Sincerely,

Dr. Tiroyaone Brombacher
